# Transcriptome profiling of root microRNAs reveals novel insights into taproot thickening in radish (*Raphanus sativus* L.)

**DOI:** 10.1186/s12870-015-0427-3

**Published:** 2015-02-03

**Authors:** Rugang Yu, Yan Wang, Liang Xu, Xianwen Zhu, Wei Zhang, Ronghua Wang, Yiqin Gong, Cecilia Limera, Liwang Liu

**Affiliations:** National Key Laboratory of Crop Genetics and Germplasm Enhancement; Engineering Research Center of Horticultural Crop Germplasm Enhancement and Utilization, Ministry of Education of P.R.China; College of Horticulture, Nanjing Agricultural University, Nanjing, 210095 P.R. China; School of Life Sciences, Huaibei Normal University, Huaibei, Anhui 235000 P.R. China; Department of Plant Sciences, North Dakota State University, Fargo, ND 58108 USA

**Keywords:** *Raphanus sativus*, Taproot, Thickening, microRNA, Solexa sequencing

## Abstract

**Background:**

Radish (*Raphanus sativus* L.) is an economically important root vegetable crop, and the taproot-thickening process is the most critical period for the final productivity and quality formation. MicroRNAs (miRNAs) are a family of non-coding small RNAs that play an important regulatory function in plant growth and development. However, the characterization of miRNAs and their roles in regulating radish taproot growth and thickening remain largely unexplored. A Solexa high-throughput sequencing technology was used to identify key miRNAs involved in taproot thickening in radish.

**Results:**

Three small RNA libraries from ‘NAU-YH’ taproot collected at pre-cortex splitting stage, cortex splitting stage and expanding stage were constructed. In all, 175 known and 107 potential novel miRNAs were discovered, from which 85 known and 13 novel miRNAs were found to be significantly differentially expressed during taproot thickening. Furthermore, totally 191 target genes were identified for the differentially expressed miRNAs. These target genes were annotated as transcription factors and other functional proteins, which were involved in various biological functions including plant growth and development, metabolism, cell organization and biogenesis, signal sensing and transduction, and plant defense response. RT-qPCR analysis validated miRNA expression patterns for five miRNAs and their corresponding target genes.

**Conclusions:**

The small RNA populations of radish taproot at different thickening stages were firstly identified by Solexa sequencing. Totally 98 differentially expressed miRNAs identified from three taproot libraries might play important regulatory roles in taproot thickening. Their targets encoding transcription factors and other functional proteins including *NF-YA2*, *ILR1*, *bHLH74, XTH16*, *CEL41* and *EXPA9* were involved in radish taproot thickening. These results could provide new insights into the regulatory roles of miRNAs during the taproot thickening and facilitate genetic improvement of taproot in radish.

**Electronic supplementary material:**

The online version of this article (doi:10.1186/s12870-015-0427-3) contains supplementary material, which is available to authorized users.

## Background

Radish (*Raphanus sativus* L*.*, 2n = 2x = 18) is an economically important root vegetable crop belonging to the Brassicaceae family [1]. The fleshy taproot comprises the main edible portion of the plant. Therefore, the taproot thickening phase is a critical period of root development that mainly determines yield and quality in radish. During taproot thickening process, an abundance of storage compounds and secondary metabolites are synthesized, which mainly determine the economic value of radish taproot and provide nutrients and medicinal function for human beings [2]. It is therefore of significance to clarify the molecular genetic mechanism underlying taproot thickening in radish.

The fleshy taproot thickening of radish is a complex biological process involving morphogenesis and dry matter accumulation [1]. Previous studies of the taproots have been focused mainly on the morphological and physio-biochemical levels. For example, the taproot axis of radish is composed of the hypocotyl and true root tissue [3], and the thickening of taproot was mainly due to the activity of a vascular cambium and the differentiation of secondary xylem and phloem [3,4]. Additionally, some studies have demonstrated that taproot development in radish was controlled by complex interactions among genetic, environmental and physiological factors [1,5]. However, root development and response to the environment are thought to be controlled by gene regulatory networks [6]. To date, great advances about gene regulation in root development have been made in several plant species [7], such as *Arabidopsis thaliana* [6,8], *Zea mays* [9], and *Oryza sativa* [10]. Unlike other roots, the taproot of radish is a storage root, the knowledge about gene regulation and the molecular mechanism is little known in storage root development, including radish. Recently, radish genome sequencing and the radish root transcriptomics studies have facilitated the investigation of the molecular mechanisms in radish taproot development [11,12]. Nevertheless, the key gene isolation and molecular mechanism underlying radish taproot thickening remain elusive.

MicroRNAs (miRNAs) are class of important non-protein-coding regulatory small RNAs (20 to 24 nt) that mediate gene expression at transcriptional and post-transcriptional level by repressing gene translation or degrading target mRNAs [13-16]. During the last decades, miRNAs have been discovered as regulators of numerous physiological and developmental processes during the life cycle of plants, including root development. For example, in *Arabidopsis*, miR164 targets NAC domain containing protein 1 (*NAC1*) to regulate lateral root development [14]; miR169 isoform targets nuclear transcription factor Y subunit A (*NF-YA*) to regulate primary root growth [15]; miR160 is involved in adventitious rooting and root cap development through the regulation of auxin response factors (*ARFs*) [16].

Recently, high-throughput sequencing technology has become a valuable tool to discover a large set of diverse plant miRNAs. Up to now, a large number of miRNAs in different plant species have been registered in miRBase 21.0 database (http://www.mirbase.org/cgi-bin/browse.pl). Additionally, several studies using this approach have identified some miRNAs and explored the roles of miRNAs in root development in *Medicago truncatula* [17], maize [18,19], rice [20] and potato [21]. In maize, 246 conserved, 32 novel and some dramatically differentially expressed miRNAs were identified in different maize roots [18]. Additionally, 137 known and 159 novel miRNAs, and 30 differentially expressed miRNAs, as well as 15 target genes, were identified during the early development of the maize brace root [19]. As one of the most important root vegetable crop, the regulatory roles of microRNAs in radish have been extensively studied in recent years. Some conserved miRNAs and novel miRNAs were identified from radish roots based on the *R. sativus* EST and GSS sequences [22,23]. Although a significant fraction of miRNAs associated with some important agronomic traits including cadmium (Cd) accumulation and embryogenesis have been successfully identified in radish [24,25], there is as yet no report on the characterization of miRNAs and their roles in regulating taproot growth and thickening in radish. To investigate the miRNA-mediated regulatory mechanism during this process, Solexa sequencing of three small RNA libraries from ‘NAU-YH’ taproots collected at pre-cortex splitting stage (Stage1, 10 DAS), cortex splitting stage (Stage2, 20 DAS) and expanding stage (Stage3, 40 DAS) were performed, respectively. As a result, some known and new miRNA families were isolated from these three taproot libraries, from which the differentially-expressed miRNAs involved in taproot thickening were identified. Subsequently, the targets of differentially expressed miRNAs were predicted and their potential functions were discussed. In addition, expression profiling of several miRNAs and their targets were further validated by RT-qPCR technology. These results would firstly reveal the miRNA-mediated regulatory network during radish taproot thickening, and provide novel insights into the molecular genetic mechanisms underlying storage root development in radish.

## Methods

### Plant growth and sample collection

**Figure 1:**
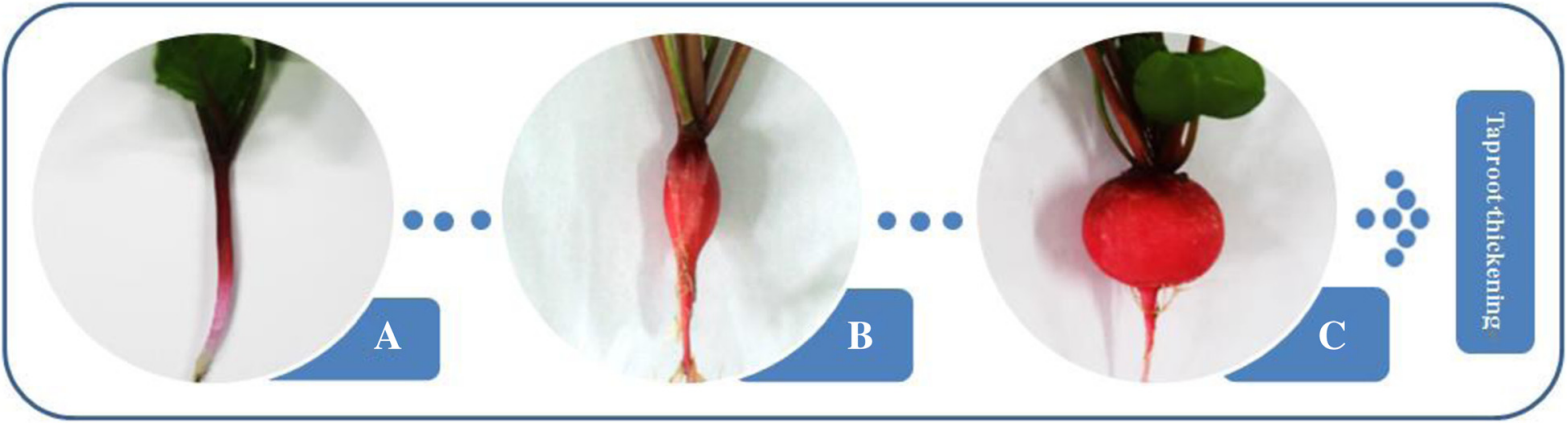
**The morphology of ‘NAU-YH’ taproot in three different thickening stages. (A)** Morphology of the pre-cortex splitting stage, 10 DAS. **(B)** Morphology of the cortex splitting stage, 20 DAS. **(C)** Morphology of the expanding stage, 40 DAS.

The radish (*Raphanus sativus* L.) advanced inbred line ‘NAU-YH’ was used in this study. Seeds were germinated on moist filter paper in darkness for 3 d, and then transplanted into plastic pots with mixture of soil and peat substrate (1:1, V/V), and cultured in the greenhouse. Samples of taproots were collected at three different development stages: pre-cortex splitting stage (Stage1, 10 DAS), cortex splitting stage (Stage2, 20 DAS) and expanding stage (Stage3, 40 DAS). Taproot developmental stages of ‘NAU-YH’ were identified using the established morphological traits (Figure 1). The subsamples of taproots were collected from five developmental stages: 10, 15, 20, 40, and 50 DAS, respectively, for RT-qPCR verification. All samples were snap-frozen in liquid nitrogen and stored at −80°C for further use.

### Transcriptome and small RNA sequencing

Total RNA was extracted from the taproot of ‘NAU-YH’ at pre-cortex splitting stage (stage1), cortex splitting stage (stage2), and expanding stage (stage3) using Trizol regent (Invitrogen, USA) following the manufacturer’s protocol. Equal amounts of total RNA from the three samples were mixed to construct a transcriptome library using an Illumina TruSeq RNA Sample PrepKit following the manufacturer’s instructions. After removing sequence reads containing low-quality sequences (reads with ambiguous bases ‘N’), adapter sequences, and reads with more than 10% Q<20 bases, mRNA transcriptome *de novo* assembly was performed using the Trinity program [26].

The extracted RNA from the taproot samples of three thickening stages were respectively used for three small RNA libraries construction including stage1, stage2 and stage3. Small RNAs of 18–30 nt in length were separated and purified by denaturing polyacrylamide gel electrophoresis. After dephosphorylation and ligation of a pair of Solexa adaptors to their 5′ and 3′ends, the products were reverse-transcribed and amplified by RT-PCR. Both the paired-end transcriptome and sRNA sequencing were performed at the Beijing Genomics Institute (BGI)-Shenzhen, China.

### Data analysis

After Solexa sequencing, the clean reads were obtained from raw reads by getting rid of the contaminated reads including sequences with 5′-primer contaminants, and poly(A) tails, without 3′-primer and the inserted tag, either shorter than 18 nt or longer than 30 nt. Then the unique RNAs were aligned with the radish reference sequences including the mRNA transcriptome sequences, EST sequences (http://www.ncbi.nlm.nih.gov/nucest/?term=radish) and genomic survey sequences (GSS, ftp://ftp.ncbi.nlm.nih.gov/repository/UniGene/Raphanus_sativus/Rsa.seq.uniq.gz) using SOAP2 program [27]. Sequences ranging from 18 to 30 nt (reads with no “N”, no more than 4 bases with quality score <10 and no more than 6 bases with quality score <13) were collected for further analysis. Firstly, the sequences matching non-coding RNAs [tRNAs, rRNAs, small nucleolar RNAs (snoRNAs) and small nuclear RNAs (snRNAs)] deposited in the Rfam 10.1 (http://www.sanger.ac.uk/Software/ac.uk/Software/Rfam) and NCBI GeneBank databases (http://www.ncbi.nlm.nih.gov/GenBank/) were eliminated. Then, using a BLASTn search, the remaining sequences with a maximum of two mismatches mapped onto known plant mature miRNAs in miRBase 21.0 (http://www.mirbase.org/index.shtml) were considered as known miRNAs.

The remaining unannotated sRNAs were used to predict novel miRNA using Mireap software (https://sourceforge.net/projects/mireap/), and the stem-loop structure of miRNA precursor was constructed by M-fold program [28]. Basic criteria by Meyers et al. (2008) and Kong et al. (2014) were used for identifying the potential novel miRNA candidates [18,29].

### Differential expression analysis of miRNAs in three libraries

To identify the differentially expressed miRNAs among three different taproot thickening stages, the miRNA expression profiles among three sRNA libraries (stage1 versus stage2; stage1 versus stage3; stage2 versus stage3) were comprehensively compared. The clean read of the tag for each miRNA was normalized to one million [25]. After normalization, if the expression level was less than one between two libraries, differential expression analysis was not performed owing to their too low expression level; if the normalized read count of a given miRNA is zero, the expression value is set to 0.01 for further analysis.

The differentially expressed miRNAs were screened with a threshold of fold change ≥ 1.0 or ≤ −1.0 (the log_2_ treatment/control) and with *P*-value < 0.05 at stage2 and stage3 versus stage1, and stage3 versus stage2, where stage1 and stage2 served as the control, respectively. The *P*-value was calculated according to previously described by Li et al. [27]. Candidate targets of differentially expressed miRNAs were predicted by aligning the miRNA sequences with the available radish reference sequences (GSS, EST and our mRNA transcriptome sequences) using the plant small RNA target analysis server (psRNATarget) with default parameters [30]. The KOBAS 2.0 program (http://kobas.cbi.pku.edu.cn/home.do) and Blast2GO program (http://www.blast2go.com/) were used to annotate the functions of the potential target sequences [25].

### RT-qPCR validation of miRNAs and their potential targets

Total RNA were isolated from the five taproot samples (10, 15, 20, 40 and 50 DAS, respectively) using Trizol reagent (Invitrogen, USA) and then treated with PrimeScript® RT reagent Kit (Takara, Dalian, China) to reverse transcribe into cDNA. MicroRNA was extracted from five radish taproot samples using RNAiso for small RNA kit (Takara, Dalian, China) and reverse transcribed into cDNA using a One Step PrimeScript® miRNA cDNA Synthesis Kit (Takara, Dalian, China). The cDNA was quantified by an iCycler IQ real-time PCR detection system (BIO-RAD) using a 20 μl reaction mixture, which consisted of 2 μl of diluted cDNA, 0.2 μM forward and reverse primer, and 10 μl of 2× SYBR Green PCR Master Mix (Takara, Dalian, China). The amplification reaction for miRNAs and their targets was performed, respectively, according to the previous reports [24,25,31]. The equation ratio 2^−ΔΔ*C*τ^ was applied to calculate the relative expression level of miRNAs and targets using 5.8S rRNA and *Actin* gene as the reference gene, respectively. The primers for real-time RT-qPCR were designed using Beacon Designer 7.0 software (Additional file 1A and B). In addition, the statistical analysis with SAS Version 9.0 software (SAS Institute, Cary, North Carolina, USA) was performed using Duncan’s multiple range test at the *P* < 0.05 level of significance.

## Results

### Root transcriptome and small RNA sequencing

A total of 51.2 million clean reads were generated in the transcriptome sequencing. By trinity assembly, totally 130,953 contigs with a mean length of 352 nt and 70,168 unigenes with an average length of 717 nt were obtained, which were then combined with the available GSS and EST sequence records in NCBI database to perfect the radish reference sequences for isolating miRNAs associated with radish taproot thickening and development.

**Figure 2:**
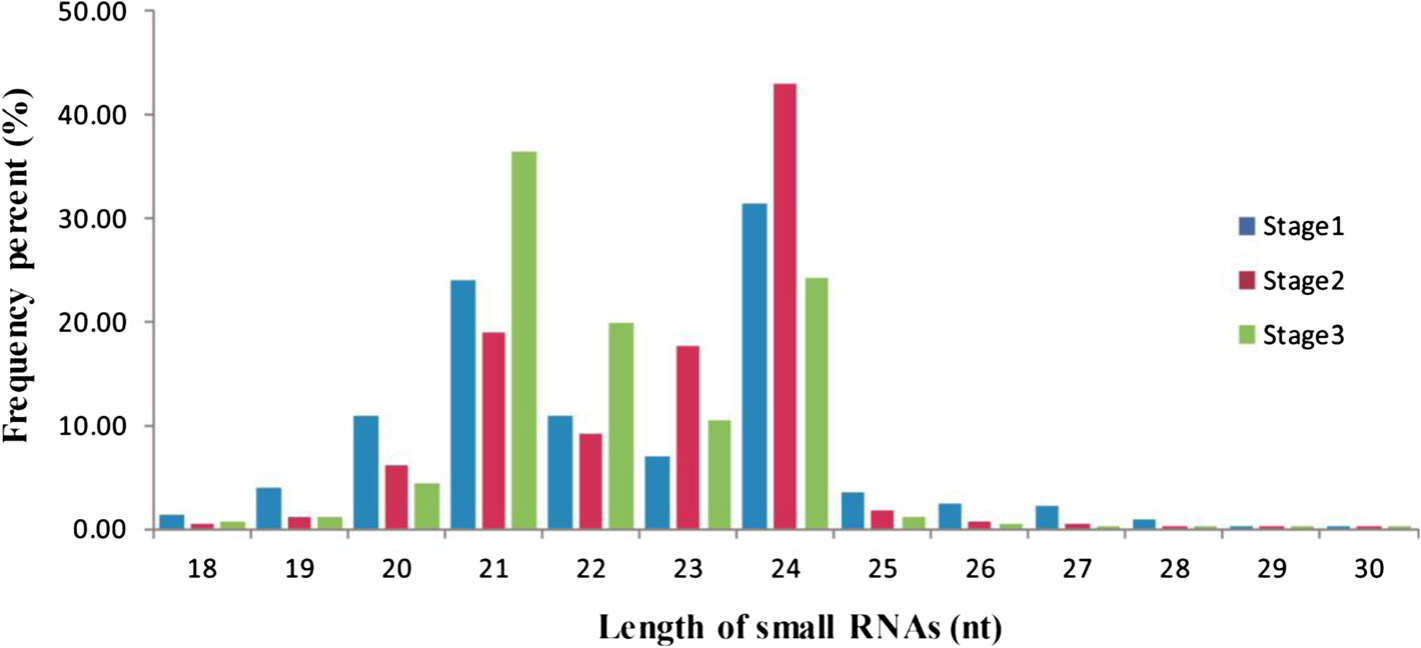
**The length distribution of small RNAs in three libraries.**

To identify miRNAs involved in radish taproot thickening and development, three small RNA libraries, stage1 (10 DAS), stage2 (20 DAS) and stage3 (40 DAS), were constructed, and then sequenced by the Illumina Solexa system. As a result, 17,160,426 (stage1), 19,055,129 (stage2) and 17,263,334 (stage3) raw reads were generated, respectively (Table 1). After removing low-quality reads and trimming adaptor sequences, 16,819,905 clean reads (4,318,929 unique) for stage1, 18,853,348 clean reads (6,575,007 unique) for stage2 and 17,082,616 clean reads (4,542,390 unique) for stage3 were obtained for further analysis (Additional file 2). Among these clean reads, comparative analysis of the common and specific reads of sRNAs between random two libraries, more than 60% of the total sRNAs were common to two different libraries, while the unique sequence reads were common only accounted for small fraction (10%–13%), indicating that there was a less abundant but variety pool of stage-specific small RNAs (Additional file 3A-F). The length of most of sRNA reads (18 to 30 nt) were 21 to 24 nt in these three stages (Figure 2). In stage1 and stage2 library, the highest proportion (>31.53%) of sRNAs was 24 nt in length, followed by 21 nt (>19.03%), which was consistent with previous studies in other species such as *M. truncatula* [17], maize [18] and potato [21]. However, the highest proportion (36.53%) of sRNAs was 21 nt in stage 3 library, followed by 24 nt (24.16%). This result was also observed in grape, in which the number of 21 nt sequence reads were more than five times of 24 nt reads [32]. Overall, these results suggest the existence of complex and diverse sRNA populations in radish.

**Table 1:** **The result of sRNA sequences from three libraries**

**Category**	**Stage1**		**Stage2**		**Stage3**	
	**Count**	**Percentage**	**Count**	**Percentage**	**Count**	**Percentage**
**Raw reads**	17160426		19055129		17263334	
**High-quality**	17087884	100%	18975347	100%	17190771	100%
**3′ adapter null**	2578	0.02%	2945	0.02%	2380	0.01%
**Insert null**	2292	0.01%	1415	0.01%	1190	0.01%
**5′ adapter contaminants**	152282	0.89%	83280	0.44%	56475	0.33%
**Smaller than 18 nt**	104474	0.61%	31266	0.16%	46772	0.27%
**Poly (A)**	6353	0.04%	3093	0.02%	1338	0.01%
**Clean reads**	16819905	98.43%	18853348	99.36%	17082616	99.37%

A total of 725,181 (stage1), 858,371 (stage2) and 906,459 (stage3) unique sequences were successfully mapped to the radish reference sequences, respectively (Additional file 2). Subsequently, for annotation, the acquired sRNA sequences were matched with NCBI GenBank, Rfam, and miRbase 21.0 database. The non-coding sRNAs were classified into six categories including miRNA, rRNAs, snRNAs, snoRNAs, tRNAs and those detected but without annotation (Additional file 2). Of all the sRNA categories, un-annotated sRNAs accounted for an average of 69.96% in total acquired sRNAs (Additional file 2). There were large variations about the number of matching unique miRNAs in these three different stages of taproot thickening, 18,078 (stage1), 36,239 (stage2) and 23,604 (stage3) unique miRNAs reads were matched to known miRNAs, respectively, implying that miRNA-mediated gene silencing is involved in the regulation of radish taproot thickening.

### Identification of known miRNAs during radish taproot thickening

To identify the known miRNAs, the small RNA sequences were mapped with known mature miRNAs from plants in miRBase 21.0 with a maximum of two mismatches. A total of 175 known miRNAs (148, 150 and 141 in the stage1, stage2 and stage3 libraries, respectively) from 57 families were detected during the radish taproot thickening process (Additional file 4A, B, C and D). Among these miRNAs, 120 (68.57%) known miRNAs were detected in all three libraries, while 145 miRNAs were shared in at least two of three small RNA libraries, and only 30 miRNAs (16, 7 and 7 in the stage1, stage2 and stage3 libraries, respectively) were stage-specifically expressed, implying that the component of miRNAs during taproot thickening was relatively stable (Additional file 3G). In this study, 144 know miRNA sequences belonging to 31 conserved miRNA families were confirmed (Additional file 4D). For example, miR156, miR158, miR159, miR160, miR167, miR394 and miR398 are conserved in a variety of plant species (Table 2 and Additional file 4D). Of these, several miRNAs, such as miR156, miR158, miR159, miR160, miR166, miR168 and miR2118, were expressed at relatively high levels, suggesting that they are highly expressed in root and possibly important regulators for radish root development. In addition, it could be found that 31 known miRNA sequences representing 26 non-conserved miRNA families, such as miR400, miR774, miR812, miR825, miR831, miR1510, miR3630 and miR8005, were previously identified only from one or few plant species.

**Table 2:** **Summary information of known miRNA families and their transcript abundance identified in all libraries**

**miRNA family**	**No. of members**	**miRNA reads**			**Normalized read count**			**Fold change**		
		**Stage1**	**Stage2**	**Stage3**	**Stage1**	**Stage2**	**Stage3**	**Log**_**2**_ **(stage2/stage1)**	**Log**_**2**_ **(stage3/stage1)**	**Log**_**2**_ **(stage3/stage2)**
**Conserved miRNA**										
miR156	19	1255950	197473	60169	30069.08	2684.56	950.56	−3.49	−4.98	−1.50
miR158	2	557972	982854	368856	20520.33	27460.11	11365.77	0.42	−0.85	−1.27
miR159	11	57202	53482	8715	16.29	0.01	6.67	−10.67	−1.29	9.38
miR160	5	1871124	1233815	437740	16.83	12.20	5.09	−0.46	−1.72	−1.26
miR161	1	113	15	0	6.72	0.80	0.01	−3.08	−9.39	−6.31
miR162	4	2343	1994	1126	0.01	0.27	0.06	4.73	2.55	−2.18
miR164	6	31856	9637	7293	1631.16	422.95	349.19	−1.95	−2.22	−0.28
miR166	14	560689	400549	158105	1040.43	643.12	276.19	−0.69	−1.91	−1.22
miR167	7	11164	13684	9386	96.43	151.64	120.71	0.65	0.32	−0.33
miR168	4	182143	183619	99034	10707.73	9669.95	5744.26	−0.15	−0.90	−0.75
miR169	10	30809	21665	18662	1.66	0.95	0.70	−0.80	−1.24	−0.44
miR171	5	158	470	51	0.01	12.46	2.87	10.28	8.16	−2.12
miR172	6	1234	6039	4154	19.03	26.31	53.21	0.47	1.48	1.02
miR390	5	22804	7150	2234	1301.37	358.88	118.37	−1.86	−3.46	−1.60
miR391	2	4090	5165	275	242.45	272.10	16.10	0.17	−3.91	−4.08
miR393	2	35	36	59	0.06	0.16	0.01	1.42	−2.57	−3.99
miR394	2	702	426	654	0.01	2.12	1.52	7.73	7.25	−0.48
miR395	3	39	50	18	1.90	0.01	0.01	−7.57	−7.57	0.00
miR396	10	2795	1792	1427	109.75	32.46	22.95	−1.76	−2.26	−0.50
miR397	1	2316	172	30	137.69	9.12	1.76	−3.92	−6.29	−2.38
miR398	3	804	1638	28150	47.50	86.46	0.01	0.86	−12.21	−13.08
miR399	4	65	87	26	0.01	0.05	0.18	2.41	4.13	1.73
miR403	1	2175	1604	1053	129.31	85.08	61.64	−0.60	−1.07	−0.46
miR408	3	11998	3335	400	712.19	176.31	23.42	−2.01	−4.93	−2.91
miR482	3	68	2640	2324	0.01	140.03	0.01	13.77	0.00	−13.77
miR535	2	1402	2121	709	0.65	0.80	0.41	0.28	−0.67	−0.96
miR824	2	4469	330	344	219.80	13.05	16.16	−4.07	−3.77	0.31
miR827	1	353	122	27	20.99	6.47	1.58	−1.70	−3.73	−2.03
miR828	2	56	18	33	3.33	0.95	1.05	−1.80	−1.66	0.14
miR2111	2	10	229	47	0.59	4.93	0.94	3.05	0.66	−2.40
miR2118	2	92829	15700	12723	2834.86	0.01	0.01	−18.11	−18.11	0.00
Total	144	4709767	3147905	1223824	280011.51	166968.59	71641.49	−0.75	−1.97	−1.22
**Non-conserved miRNA**										
miR400	1	147	72	41	8.74	4.28	2.40	−1.03	−1.86	−0.83
miR774	1	307	10640	5198	18.25	632.58	304.29	5.12	4.06	−1.06
miR812	1	0	3145	1503	0.00	186.98	87.98	18.26	17.18	−1.09
miR825	1	1675	0	0	99.58	0.00	0.00	−17.35	−17.38	−0.02
miR831	1	267	295	760	15.87	17.54	44.49	0.14	1.49	1.34
miR845	2	11735	7945	1722	697.69	472.36	100.80	−0.56	−2.79	−2.23
miR858	2	22	28	153	1.31	1.66	3.40	0.35	1.38	1.03
miR859	1	444	956	347	26.40	56.84	20.31	1.11	−0.38	−1.48
miR862	1	76	224	58	4.52	13.32	3.40	1.56	−0.41	−1.97
miR1310	1	1376	273	296	81.81	16.23	17.33	−2.33	−2.24	0.09
miR1510	2	1419	0	1905	84.36	0.00	111.52	−17.11	0.40	17.52
miR1511	2	14510	22556	8500	862.67	1341.03	497.58	0.64	−0.79	−1.43
miR1513	1	484	1001	0	28.78	59.51	0.00	1.05	−15.59	−16.63
miR1520	1	36042	7457	6131	2142.82	443.34	358.90	−2.27	−2.58	−0.30
miR1885	1	3846	1383	1444	228.66	82.22	84.53	−1.48	−1.44	0.04
miR3630	2	266	52	0	15.81	3.09	0.00	−2.35	−14.72	−12.37
miR4378	1	0	9787	1082	0.00	581.87	63.34	19.90	16.70	−3.20
miR5654	1	8098	2411	738	481.45	143.34	43.20	−1.75	−3.48	−1.73
miR5763	1	0	370	669	0.00	22.00	39.16	15.18	16.01	0.83
miR5774	1	0	0	1234	0.00	0.00	72.24	0.00	16.89	16.89
miR6164	1	892	971	615	53.03	57.73	36.00	0.12	−0.56	−0.68
miR7504	1	45	113	5348	2.68	6.72	313.07	1.33	6.87	5.54
miR7510	1	218	383	171	12.96	22.77	10.01	0.81	−0.37	−1.19
miR7532	1	8125	2752	2304	483.06	163.62	134.87	−1.56	−1.84	−0.28
miR8005	1	0	1118	0	0.01	59.2998	0.01	12.53	0.00	−12.53
miR8041	1	18	62	27	1.07	3.69	1.58	1.78	0.56	−1.22
Total	31	90012	73994	40246	5351.52	3865.41	3469.08	−0.47	−0.63	−0.16
Total	175	4799779	3221899	1264070	285363.03	170834.01	75110.57	−0.74	−1.93	−1.19

**Figure 3:**
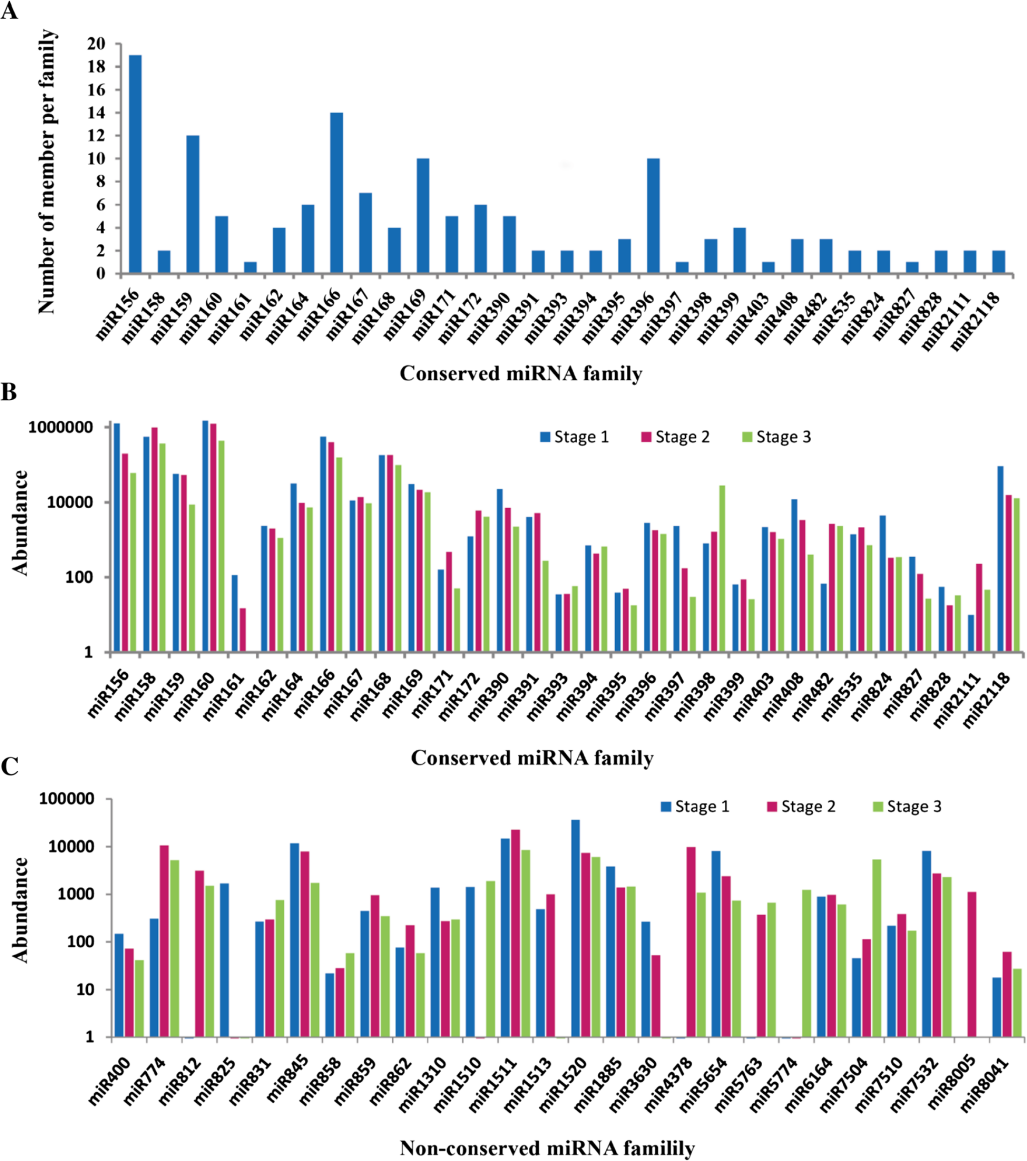
**Sizes and abundance of identified known miRNA families in radish.** The distribution of conserved miRNA family size **(A)** and the abundance of conserved **(B)** and non-conserved **(C)** miRNA family.

Furthermore, the members of known miRNA families were also analyzed in this study. Among conserved miRNA families (Figure 3A), the miR156 was the largest family with 19 members, followed by miR166, miR159, miR169 and miR396, with 14, 11, 10 and 10 members, respectively. Of remaining 26 miRNA families, 22 comprised two to seven members, and others had only one member (Table 2). In addition to the conserved miRNA families, the other 26 non-conserved miRNA families comprised only one or two members (Table 2). The expression levels of known miRNA families were also analyzed. Among the 31 conserved miRNA families, the expression levels of several miRNA families including miR158, miR160 and miR166 showed high abundance, each with total read >100,000 (Figure 3B). In contrast, very low level of expression was found in some miRNA families including miR161, miR395 and miR828. Meanwhile, 31 conserved miRNA families were also found to be more abundant than non-conserved miRNAs (Table 2, Figure 3B and C). In addition, various members within the same family showed considerably variable in expression levels, for example, the number of miR156 family member reads ranged from one to 719,515 in three libraries (Additional file 4D). Moreover, the same member within different stages also indicated different read numbers, for instance, the abundance of miR156a in stage1, stage2 and stage3 were 505,759, 50,613 and 16,238 reads, respectively, implying that there were various functional divergences within miRNA family during the radish taproot thickening.

### Identification of novel miRNA candidates during radish taproot thickening

Based on the key characteristics of novel miRNA [18,29], the formation of stem loop structure of precursor is prerequisite for a new miRNA. In total, 107 potential novel miRNAs (90 miRNA families) were predicted from three libraries (Additional file 5A). The stem loop structures of these predicted miRNA precursors were shown in Additional file 6. In addition to stem-loop structure prediction, detection of complementary sequences is another way to increase the authenticity of predicted novel miRNAs [29]. Among these potential novel miRNAs, five potential novel miRNA with complementary sequences were detected as the novel miRNA candidates (Additional file 5B).

In this study, the predicted hairpin length of these 107 potential novel miRNA precursors ranged from 47 to 354 nt. The folding of minimum free energy (MFE) value of these miRNA precursors ranged from −18.3 to −95.2 kcal/mol with an average of −40.1 kcal/mol (Additional file 5A). In addition, only seven out of 107 predicted miRNAs candidates were shared by all three libraries, while 53, 73 and 39 miRNAs were detected in stage1, stage2 and stage3 libraries, respectively (Additional file 5A).

The 107 potential novel miRNAs exhibited lower expression levels with the abundance ranging from five to 3,318 reads, as compared with known miRNAs. In addition, the numbers of all novel complementary miRNAs reads ranging from five to 114 were clearly less than those for their corresponding mature miRNAs, which was consistent with the idea that miRNA* strands were degraded rapidly during the biogenesis of mature miRNAs [33]. Interestingly, rsa-nmiR2-5p (read count of 17 vs. 14 in stage2 library) and rsa-miR18-5p (read count of 20 vs. 11 in stage2 library) showed similar abundance between novel miRNA and complementary miRNA (Additional file 5B), indicating that both the miRNA and their complementary miRNA might be functional in regulating gene expression during the taproot thickening process in radish.

### Differentially expressed miRNAs during radish taproot thickening

**Figure 4:**
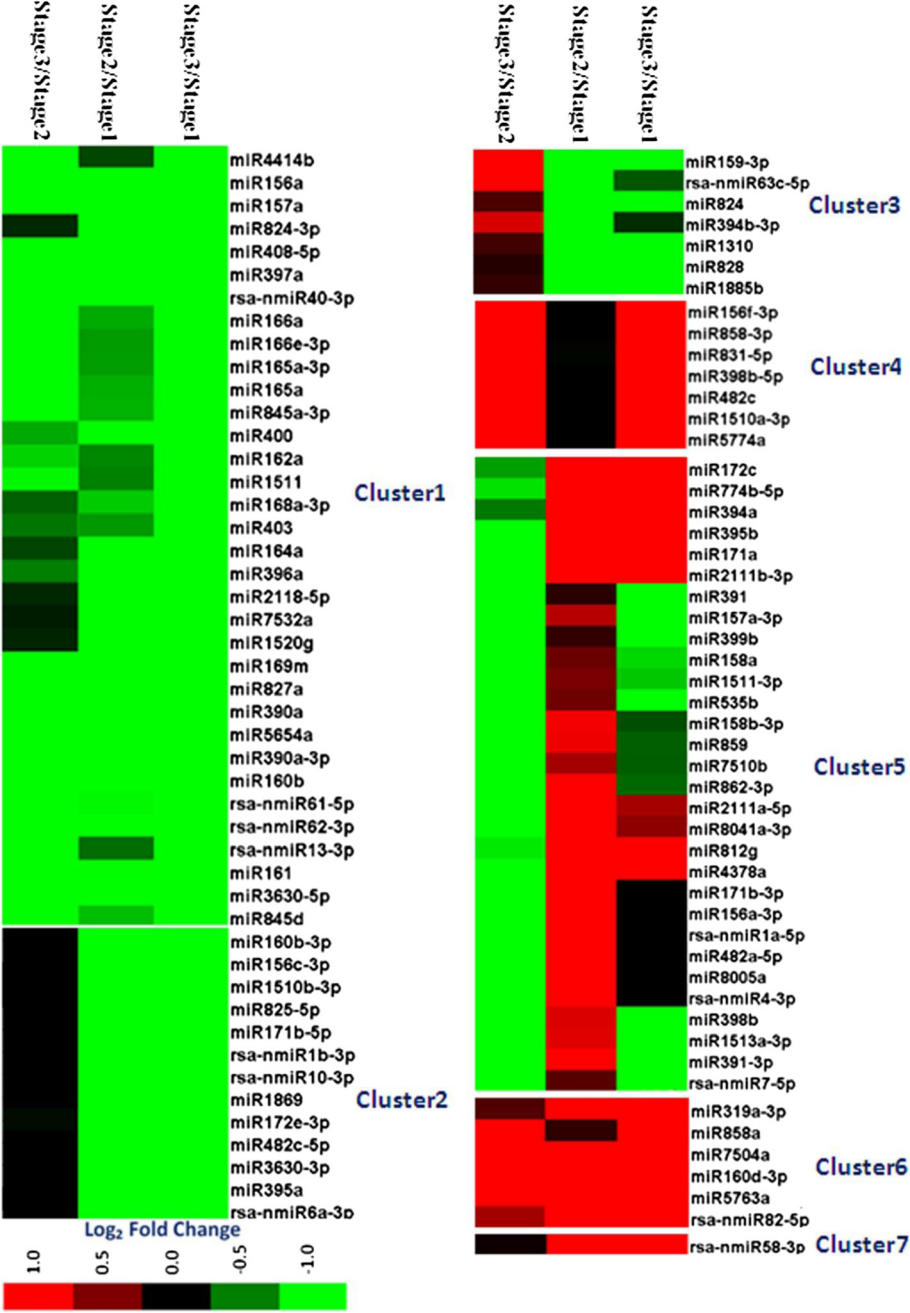
**Clustering of differentially expressed miRNAs in three libraries.** The bar represents the scale of relative miRNA expression (Log_2_ Fold change).

Differential expression analysis was performed to identify differentially expressed miRNAs during the taproot thickening process. Based on the selected criteria (At least one comparison has a fold change log_2_ scale value ≥ 1.0 or ≤ −1.0 with *P*-value < 0.05), in all, 85 known miRNAs and 13 novel miRNAs were identified as differentially expressed miRNAs (Additional file 7). It was shown here that two important transitions (Stage1 to Stage2/Stage2 to Stage3) were analyzed during taproot thickening (Additional file 8). The differentially expressed miRNAs were divided into seven clusters according to their highly similar expression patterns at the different stages of taproot thickening (Additional file 8 and Figure 4). The results indicated that 34 miRNAs had a down-regulated pattern during taproot thickening (Cluster 1 in Additional file 8). As from stage1 to stage2, the expression of 42 miRNAs including miR156a, miR157a, miR160b, miR169m, miR390a and miR397a, declined obviously (Clusters 1, 2 and 3 in Additional file 8), whereas 13 miRNAs in Cluster 1 exhibited a gradually decline. As from stage2 to stage3, 64 miRNAs exhibited down-regulated pattern (Clusters 1 and 5 in Additional file 8). In contrast, six miRNAs had an up-regulated pattern during taproot thickening (Cluster 6 in Additional file 8). The expressions of 37 miRNAs increased from stage1 to stage2 (Clusters 5, 6 and 7 in Additional file 8), and 20 miRNAs increased from stage2 to stage3 (Clusters 3, 4 and 6 in Additional file 8). Moreover, some miRNAs were preferentially expressed in only one taproot thickening stage. For example, rsa-nmiR6a-3p and rsa-nmiR4-3p were enriched at stage1 and stage2, respectively (Clusters 2, 4 and 5 in Additional file 8). Additionally, the miRNAs in Cluster 3 decreased obviously from stage1 to stage2, but increased from stage2 to stage3, whereas the miRNAs in Cluster 5 increased from stage1 to stage2, and decreased from stage2 to stage3.

**Figure 5:**
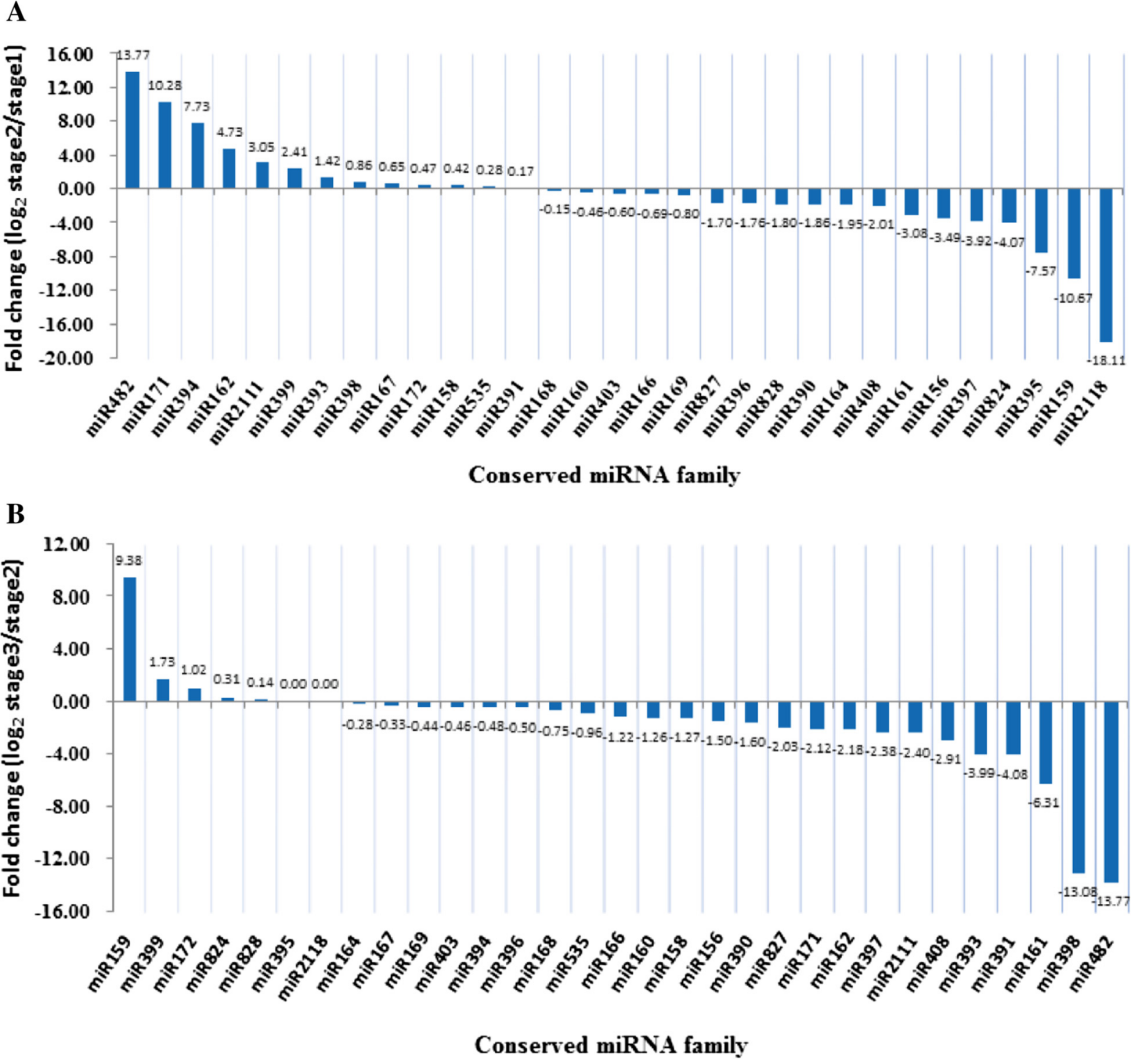
**Comparatively relative expression of differentially expressed conserved miRNA family in radish.** Comparison of stage 1 and stage 2 **(A)**, and comparison of stage 2 and stage 3 **(B)**.

Among the 31 conserved miRNA families, 13 and 18 miRNA families were up and down-regulated at stage2 as compared with stage1, respectively (Figure 5A, Table 2). Meanwhile, five and 24 miRNA families were up and down-regulated in stage3 compared with stage2, respectively (Figure 5B, Table 2). Of these, five miRNA families were differentially expressed at a ratio greater than 10-fold (Figure 5). These results implied that these miRNA sequences and miRNA families might play essential regulatory roles during radish taproot thickening.

### Prediction of potential target genes of differentially expressed miRNAs

To further clarify biological functions of the differentially expressed miRNAs during taproot thickening process, a total of 482 target sequences for 85 differentially expressed miRNAs were predicted (Additional file 9). Among these sequences, 191 potential target genes for 78 differentially expressed miRNAs were further annotated by BLAST search against *Arabidopsis* sequences using KOBAS 2.0 program (Additional file 9). Among them, 176 and 20 target genes were predicted for 67 known and 11 novel miRNAs, respectively (Additional file 9). It could be found that there are many single miRNAs targeted multiple genes and multiple miRNAs regulated a single gene. As a result, lots of these target genes were annotated as transcription factors (TFs). For instance, miR156, miR159 and miR774 family members were identified to target the squamosa promoter-binding-like protein genes (*SPLs*). miR160 family members were identified to target the auxin response factor genes (*ARFs*) and vascular plant one zinc finger protein genes (*VOZ1*). miR172 family members were identified to target the floral homeotic protein APETALA 2 gene (*AP2*), SC35-like splicing factor 33 gene (*SCL33*) and transcription factor IIIA gene (*TFIIIA*). The targets of miR164, miR169 and miR396 family members belonged to NAC-domain containing protein genes (*NACs*), nuclear transcription factor Y subunit A-2 protein gene (*NF-YA2*) and basic helix-loop-helix transcription factor bHLH74 gene (*bHLH74*), respectively. On the other hand, some target genes were annotated as other functional proteins. For instance, glutamine synthetase gene (*GS2*), IAA-amino acid hydrolase ILR1 gene (*ILR1*), laccases gene (*LACs*), xyloglucan endotransglucosylase/hydrolase protein 16 gene (*XTH16*), alkaline/neutral invertase gene (*INV-E*), protein CLAVATA3/ESR-related 41 gene (*CLE41*), expansin A9 gene (*EXPA9*), calmodulin 7 gene (*CAM7*) and protein phosphatase 2A regulatory B subunit gene (*PP2A-B*) were identified as the targets of miR156, miR172, miR397, miR858, miR5654, miR7532, miR8005, rsa-nmiR4 and rsa-nmiR6, respectively. The sulfate adenylyltransferase gene (*APS4*) and ATP sulfurylase 1 genes (*APS1*) were targeted by miR395. Additionally, some target genes were annotated as uncharacterized and hypothetical protein.

**Figure 6:**
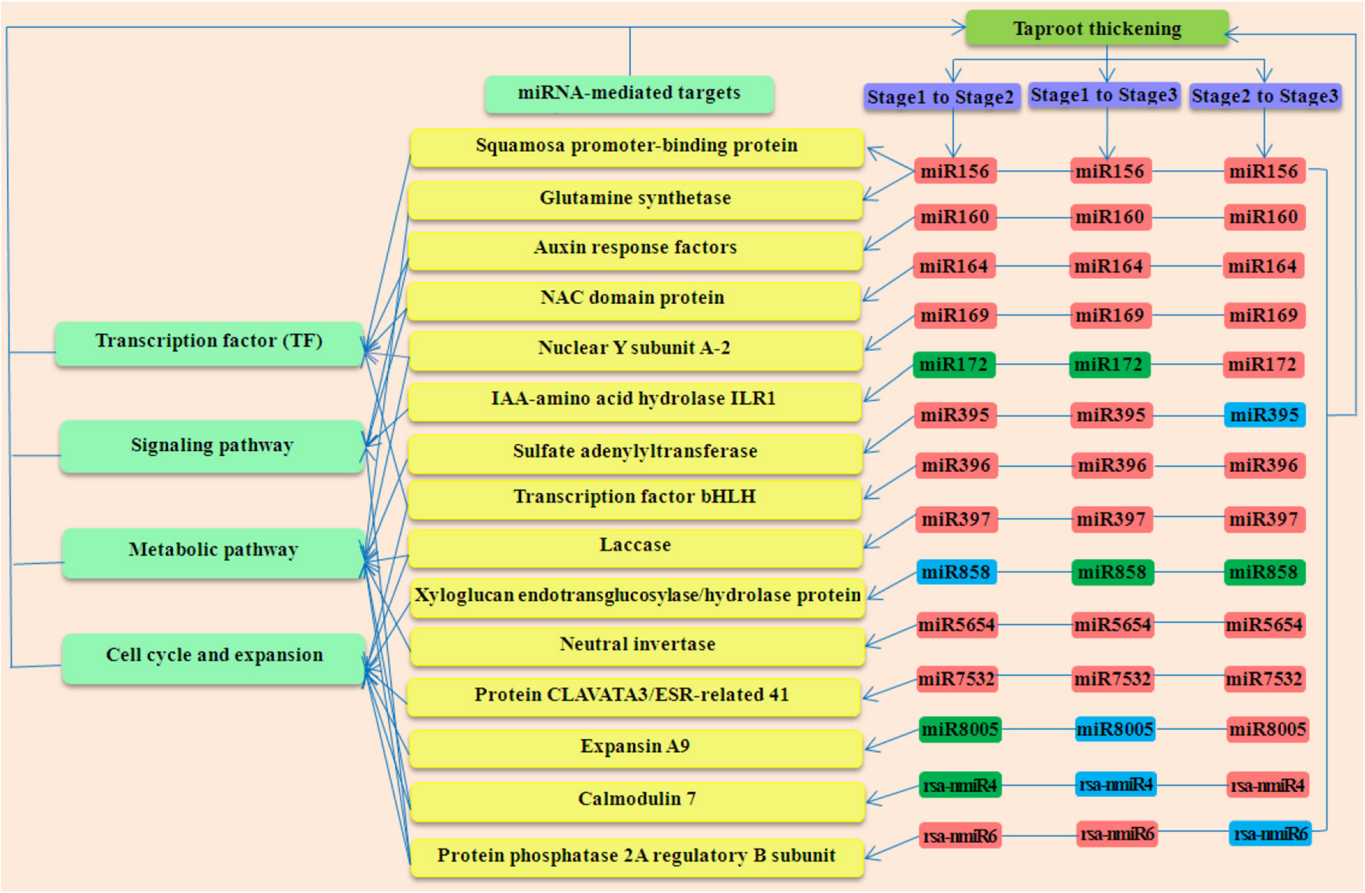
**The putative role of miRNAs during radish taproot thickening.** Green boxes: up regulated; rose-bengal boxes: down regulated; blue boxes: unchanged.

The majority of these identified target genes were annotated as transcription factors and other functional proteins which involved in plant growth and development, metabolism, cell organization and biogenesis, signal sensing and transduction, and plant defense response, such as auxin signaling, nutrition metabolism, sucrose metabolism, cell growth and cell wall expansion. All these results suggested that the differentially expressed miRNAs might play crucial regulatory roles during the taproot thickening process. The putative roles of miRNAs involved in taproot thickening of radish are summarized in Figure 6.

### GO term annotation of the differentially expressed miRNAs targets

**Figure 7:**
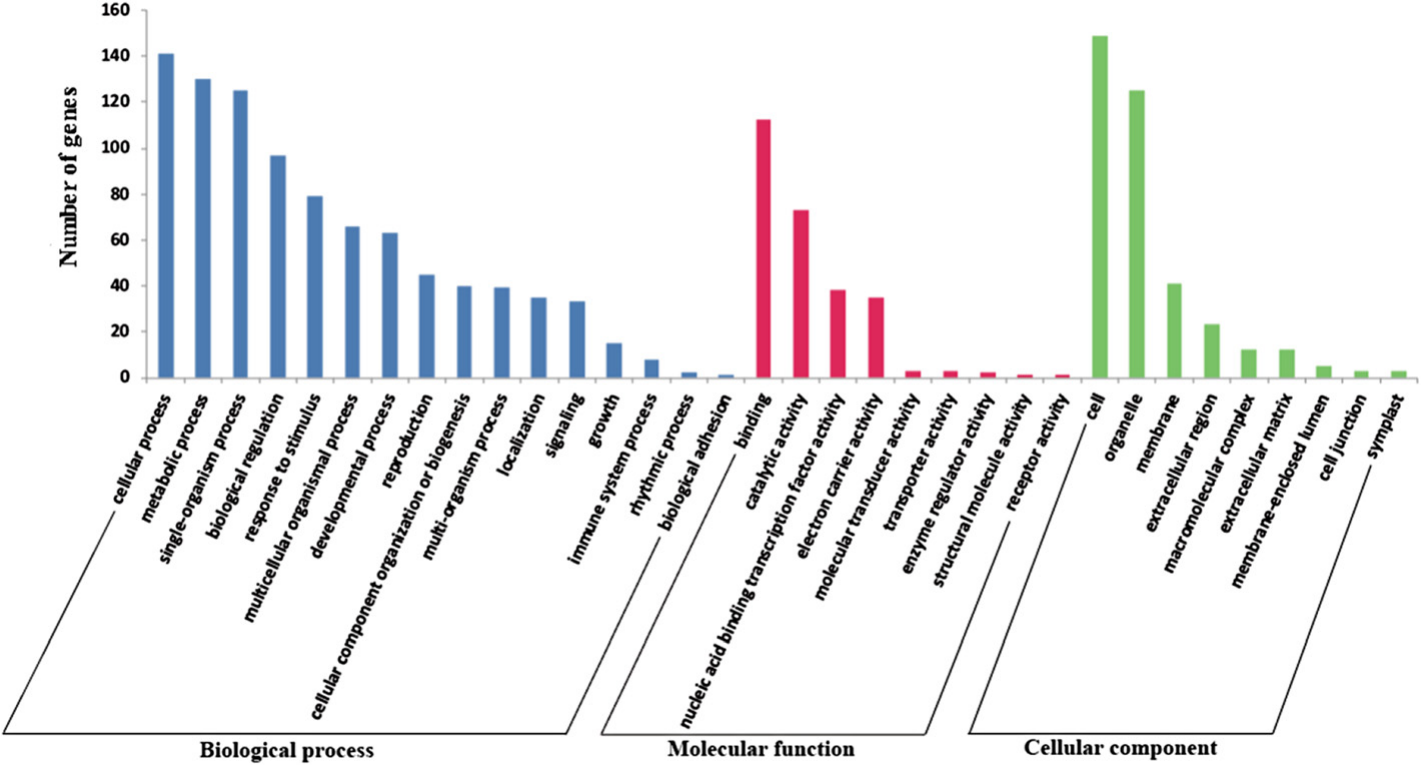
**Gene ontology of the predicted targets for differentially expressed miRNAs.**

To further investigate the function of the differentially expressed miRNAs, the predicted 191 target genes were collected to be performed a Gene Ontology (GO) term annotation using the Blast2GO program (http://www.blast2go.com) (Additional file 10). GO term annotation results indicated that 16 different biological processes, nine different molecular functions and nine different cellular components were predicted (Figure 7). Among biological process, cellular process, metabolic process, single-organism process, biological regulation, response to stimulus, multicellular organismal process and developmental process are the most significantly enriched GO terms (Figure 7). Interestingly, the enriched GO terms also showed to be involved in various development and metabolic processes, such as carbohydrate metabolic process (GO: 0005975), sucrose metabolic process (GO: 0005985), root system development (GO: 0022622), root morphogenesis (GO: 0010015) and root development (GO: 0048364) (Additional file 10). These results suggested that the taproot thickening process in three stages is potentially regulated by the miRNAs and their corresponding target genes.

### Validation of the expression patterns of differentially expressed miRNAs and their targets by RT-qPCR

**Figure 8:**
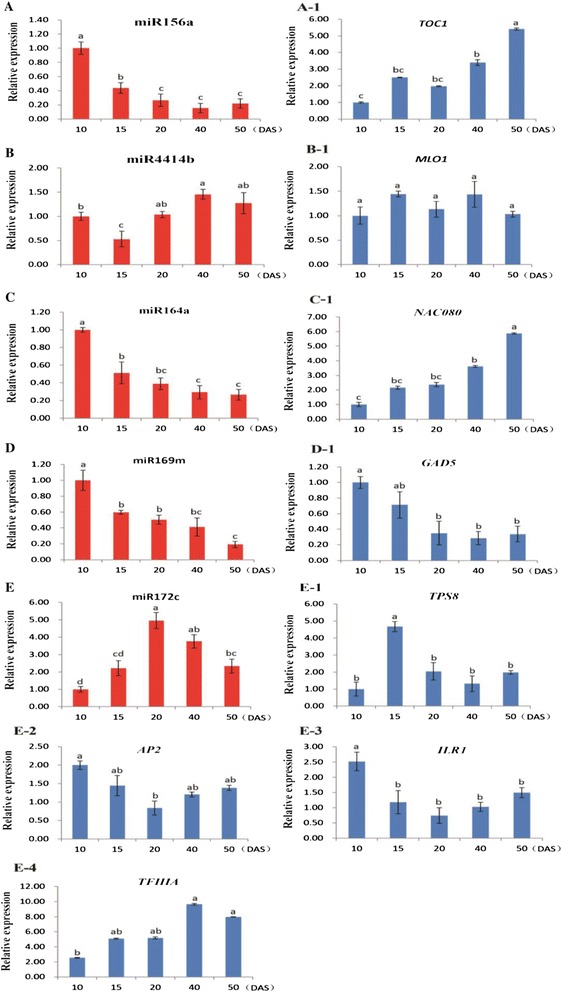
**Quantitative expression analyses of five differentially expressed miRNAs (A ~ E) and their target genes (A-1 ~ E-4).** Each bar shows the mean ± SE of triplicate assays. The values with different letters indicate significant differences at *P* < 0.05 according to Duncan’s multiple range tests.

To further confirm the expression pattern of the differentially expressed miRNAs, and their putative potential targets during radish taproot thickening process, five differentially expressed miRNAs and eight corresponding targets were randomly selected and validated with RT-qPCR. As a result, all five differentially expressed miRNAs and their targets were obviously differentially expressed among various stages of taproot development (10, 15, 20, 40 and 50 DAS) (Figure 8). Among them, miR156a, miR164a and miR169m were almost down-regulated during the taproot development (Figure 8A, C and D), while miR172c was up-regulated and peaked at 20 DAS compared with 10 DAS and 15 DAS, and then down-regulated compared with 40 DAS and 50 DAS (Figure 8E). The results showed well consistency with the expression pattern analyzed by small RNA high-throughput sequencing. However, miR4414b showed dynamic change (Figure 8B), which was not well consistent with the results of the sequencing.

Furthermore, it could be found that miRNAs and their target genes had anti-correlated expression tendencies at various taproot development stages in radish. miR156a, miR164a and miR172c as well as their corresponding target transcripts (*TOC1*, miR156a target gene, gi|167492752|gb|FD975103.1|FD975103; *NAC080*, miR164a target gene, gi|158663918|gb|EX773809.1|EX773809; *ILR1,* CL5916.Contig2_NAU-YH and *AP2,* gi|166139427|gb|FD572123.1|FD572123, miR172c target genes) had contrasting expression tendencies during various taproot thickening stages (Figure 8A-1, C-1, E-2 and E-3), suggesting that these miRNAs may regulate their potential target expressions, and the target genes of miRNAs may be involved in radish taproot thickening. However, no correlation was also observed between the expression of some miRNAs and their targets during the taproot thickening process. For instance, miR169m, miR4414b and their targets had similar expression tendency over the various taproot development stages (Figure 8B-1 and D-1), which indicating that these two predicted genes may not be targets of miR4414b and miR169m. Meanwhile, miRNA172 and its target transcript (*TFIIIA,* gi|332778718|gb|FY435119.1|FY435119) showed unique no correlation expression patterns (Figure 8E-4), suggesting that the putative target may implement a particular function during radish taproot-thickening.

## Discussion

Radish is an important root vegetable crop and its edible part is the taproot, which directly determines the yield and quality [1]. It is therefore of significance to understand the mechanism of radish taproot formation. miRNAs regulate multiple developmental events in plants. To date, much effort has been put in studying miRNA mechanisms underlying different plant development processes, such as flower development [34], seed and seedling development [35,36], and root development [19-21]. Up to now, several studies on expression profiles of miRNA associated with plant root development were conducted in many important plant species, such as maize [19], rice [20], *M. truncatula* [17] and potato [21], while an overall expression profiles of miRNA during the phase of thickening in radish taproot is still unexplored. Although some potential conserved and novel miRNAs have been predicted from radish root based on the *R. sativus* EST and GSS sequences [22,23], all of these miRNAs were obtained from taproot collected at one period, which greatly constrained the investigation of the miRNAs regulation mechanism underlying the taproot thickening. In this study, a population of both known and novel miRNAs from different thickening stages of fleshy taproot were firstly identified and characterized. Furthermore, the differentially expressed miRNAs and their potentially target genes associated with taproot thickening were also investigated in radish.

The development of cortex splitting of radish taproot marks the entry into a growth stage that mainly involves root thickening [1]. In this study, using a Solexa sequencing technology, small RNAs in developing radish taproot from three different developmental stages: pre-cortex splitting stage (Stage1, 10 DAS), cortex splitting stage (Stage2, 20 DAS) and expanding stage (Stage3, 40 DAS), which cover the key morphological changes that occur during the taproot thickening process, were firstly isolated.

### Identification of taproot thickening-related miRNAs by Solexa sequencing in radish

Since the miRNAs in Arabidopsis was firstly discovered, thousands of mature sequences in plants have been registered in miRbase, and some miRNAs were identified to be indispensable for the development and formation of plant roots [37]. Recently, by the high-throughput sequencing, 137 known and 159 novel miRNAs were obtained during the early development of the maize brace root [19], and 83 known and 24 novel miRNAs were identified in root tissues and root callus tissues in *M. truncatula* [17]. Previously, although some conserved miRNAs have been reported in radish [22-25], the miRNAs involved in the process of taproot thickening have not been discovered. In this study, 175 known miRNAs (57 miRNA families) and 107 potential novel miRNA candidates (90 miRNA families) were identified during the taproot thickening process. Among the 57 identified known miRNA families, over a half (31) are conserved with other species predicted previously [38]. Moreover, some miRNA families with high expression levels in this study are also expressed in the roots of other plant species, such as miR156, miR159, miR164, miR166, miR167, miR168 and miR172, which were abundantly found in maize brace roots [19]. These results suggested that these miRNAs may play crucial and conserved roles in plant root development. In addition, with the high-throughput sequencing technology, 107 potential novel miRNAs were identified during radish taproot thickening in this study (Additional file 5A). Among these novel miRNAs, some of them may only be detected during specific developmental stages. Compared to the conserved miRNAs, most of the novel miRNAs exhibited lower abundance levels, as previously reports indicating that the novel miRNAs were often expressed at relatively lower levels than conserved miRNAs [25]. However, although expressing at low level, these new miRNAs might play developmental-specific or species-specific roles during taproot thickening in radish.

### Dynamic expression patterns of miRNAs associated with radish taproot-thickening

Most of differentially expressed miRNAs have been identified as being involved in the regulation of plant growth and development in diverse plants [13,19,34-36]. In the present study, 85 known miRNAs belonging to 54 miRNA families, and 13 novel miRNAs belonging to 12 miRNA families were identified to be differentially expressed during radish taproot thickening (Additional file 7). Among these, more miRNAs were observed to be down-regulated than up-regulated during the taproot thickening process. For instance, miR156, miR159, miR160, miR166, miR390, miR397, miR408, miR5654, rsa-nmiR40 and rsa-nmiR62 were down-regulated significantly during the period stage1 to stage2 and stage2 to stage3. Additionally, most of these down-regulated miRNAs were also highly expressed in three libraries (Cluster 1 in Additional file 8), implying that miRNAs play vital roles during taproot thickening in radish. Meanwhile, a few miRNAs, such as miR5763 and miR7504 were increasingly up-regulated during the period stage1 to stage2 and stage2 to stage3 (Cluster 6 in Additional file 8). Some miRNAs, such as miR171, miR172, miR774, miR812, miR2111, rsa-nmiR1, rsa-nmiR4 and rsa-nmiR7, were increasingly up-regulated from stage1 to stage2, and then down-regulated from stage2 to stage3 (Cluster 5 in Additional file 8). It was reported that in plant development process, the up- or down-regulation of miRNAs might play a more important role in the regulation of network [39-41]. Therefore, it was possible that 98 miRNAs showing differential expression patterns may also play crucial roles in regulating the thickening of radish taproot, although more investigations are needed to further clarify regulation mechanism associated with various miRNAs during taproot thickening.

In addition, previous study has shown that several known miRNAs were differentially regulated during the early development of maize brace root, including six miRNA families (miR164, miR167, miR171, miR390, miR393 and miR399) were up-regulated, and two miRNA families (miR156 and miR169) were down-regulated [19]. As expected, some of these identified miRNAs also showed differential regulation during taproot thickening, such as miR156 and miR169 were down-regulated, suggesting these miRNAs may be involved in the regulatory networks during root development. Moreover, several miRNA families (miR167 and miR393) were reported to regulate root development in *Arabidopsis* [16,42], while in this study, they did not exhibit significant differences in expression during radish taproot thickening. Nevertheless, they also may be play crucial role during taproot thickening in radish, because these miRNAs expressed in all three libraries.

### miRNA-mediated regulatory networks of taproot-thickening in radish

Although many functional studies have revealed that some miRNAs play crucial roles in plant root development [16,37,42], there is few studies on characterization of miRNAs and their target genes related to storage root formation to date. In this study, 191 targets for the differentially expressed miRNAs during radish taproot thickening were found to be involved in various biological functions including plant growth and development, metabolism, cell organization and biogenesis, signal sensing and transduction, and plant defense response. Of these predicted targets, many of them are transcription factors (*SPLs*, *NF-YA2, ILR1* and *bHLH74*) and regulate hormone accumulation (*ARFs*, *NACs*), and they were identified to be involved in plant root formation and development process [14,15,43-46]. For example, *NF-YA2* transcription factor was one of the targets of miR169, which acts in the control of primary root growth in *Arabidopsis* [15]. miR156 were predicted to target mRNA coding for SPL like family transcription factor. Previous studies have proven that miR156-mediated regulation of *SPLs* involves in plant development [44]. miR160 target gene encodes auxin response factors (*ARF16, ARF17*) which were found to affect primary and lateral root growth in *Arabidopsis*, rice and *Medicago* [14,16,20,47]. In addition, some targets may be involved in regulating substances and energy metabolism changes, and are widely considered to be important for root development in plants [48]. Sulfate adenylyltransferase and ATP sulfurylase 1 (*APSs*) genes targeted by miR395 were involved in sulfur assimilation and regulated root elongation by affecting root indole-3-acetic acid levels [49]. miR5654 targeting *INV* was thought to function in regulating sucrose metabolism and it has been proven that *INV* affects the root development [50]. Moreover, a number of target genes in this study seem to be involved in regulating cell cycle and cell expansion. For instance, xyloglucan endotransglucosylase/hydrolase protein 16 (*XTH16*, targeted by miR858) and expansin A9 (*EXPA9*, targeted by miR8005) were cell wall-related genes, which regulate the extension of cell wall during plant growth [51]. Laccases (*LACs*) targeted by miR397 were associated with thickening of the cell wall in secondary cell growth [52]. The protein CLAVATA3/ESR-related 41 gene (*CLE41*) was targeted by miR7532 and controls the rate and orientation of vascular cell division [53]. The protein phosphatase 2A regulatory B subunit gene (*PP2A B subunit*) targeted by rsa-nmiR6 could regulate cell grow in root of *Arabidopsis* [54]. Calmodulin 7 gene (*CAM7*), targeted by rsa-nmiR4, was found to be involved in the cell growth-promoting pathway, for calmodulin could bind to peptide phytosulfokine (PSK) receptor to cause cell growth [55]. Additionally, among the putative taproot thickening-related miRNAs, eight miRNAs (miR156, miR160, miR164, miR169, miR396, miR397, miR5654 and miR7532) were down-regulated, and five miRNAs (miR395, miR858, miR8005, rsa-nmiR4 and rsa-nmiR6) showed stage-specific pattern of expression during the taproot thickening (Figure 6 and Additional file 8). Therefore, it could be inferred that these miRNAs and their targets play important roles in regulating radish taproot thickening.

**Figure 9:**
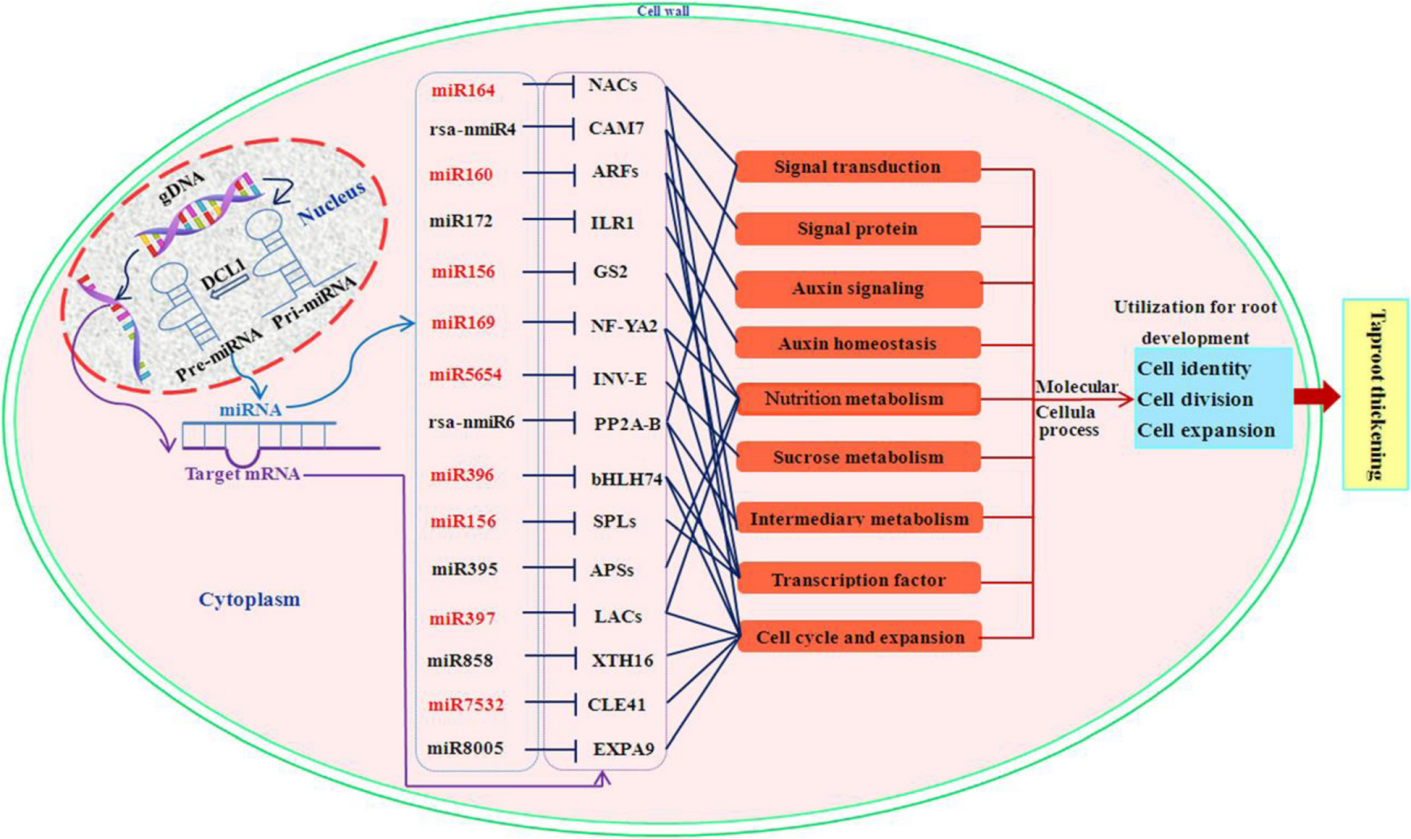
**The hypothetical model of miRNAs-mediated regulatory network associated with radish taproot thickening.** The red font represents the down-regulated miRNA in three libraries.

The thickening of taproot strongly influences the yield and quality in radish [1]. Thickening of underground storage root is a complex process with intricate pathways. Hormonal accumulation, transcription factor regulation, substances and energy metabolism changes, cell cycle and cell expansion, and others favor the thickening of taproot in radish [56-58]. In light of important functions of these differentially expressed miRNAs in regulating radish root development, a hypothetical model of miRNAs mediated regulation associated with taproot thickening in radish was put forward (Figure 9).

## Conclusions

In summary, the small RNA population of radish taproot at different thickening stages were firstly identified using Solexa sequencing technology, a total of 175 known miRNAs and 107 novel miRNAs were found to be associated with radish taproot-thickening. Totally 98 differentially expressed miRNAs (85 known and 13 novel miRNAs) were identified and their 191 target genes were engaged in various biological functions, including plant growth and development, metabolism, cell organization and biogenesis, signal sensing and transduction, and plant defense response. Gene ontology categorization and enrichment analysis of the targets corresponding to the differentially expressed miRNAs revealed that a number of miRNA-targeted genes are required for radish taproot thickening. These findings provide significant insight into miRNA-mediated molecular regulatory mechanism underlying the taproot development and formation in radish.

### Availability of supporting data

The RNA sequence dataset supporting the results of this article is available in the repository of NCBI Sequence Read Archive (SRA) with the GenBank accession No.: SRX707630.

## Additional files

Additional file 1:
**RT-qPCR validated miRNAs primer sequences (A), and targets primer sequences (B).**
Additional file 2:
**The distribution of sRNAs among different categories.**
Additional file 3:
**Venn diagrams for analysis of Small RNAs.** (A-F) Summary of common and specific unique (A, B and C) sRNAs and total (D, E and F) sRNAs between different libraries. (G) Known miRNAs among different libraries. Additional file 4:
**Detailed information of the known miRNAs identified in stage1 library (A), stage2 library (B), stage3 library (C) and in all three libraries (D).**
Additional file 5:
**Identification of novel miRNA candidates in three libraries.** (A) Potential novel miRNA candidates, (B) novel miRNA candidates with complementary sequences. Additional file 6:
**The secondary structures of novel** ***Raphanus sativus*** **miRNA precursors.**
Additional file 7:
**Expression data of miRNAs differentially expressed during radish taproot thickening.**
Additional file 8:
**The differentially expressed miRNAs in stage 1 to stage 2 and stage 2 to stage 3.**
Additional file 9:
**The potential targets of differentially expressed miRNAs during radish taproot thickening.**
Additional file 10:
**Significant GO terms for differentially expressed miRNA target genes.**

## Abbreviations

DAS: Day after sowing
RT-qPCR: Reverse transcription quantitative real-time PCR
TOC1: Timing of CAB Expression 1/two-component response regulator-like APRR1
TFIIIA: Ttranscription factor IIIA
