# Supplementary material for: Transcriptome profiling of root microRNAs reveals novel insights into taproot thickening in radish (Raphanus sativus L.)
Source: BMC Plant Biol. 2015 Feb 3;15:30. doi: 10.1186/s12870-015-0427-3 (PMC4341240; doi:10.1186/s12870-015-0427-3)
Supplement: Additional file 3: — Venn diagrams for analysis of Small RNAs. (A-F) Summary of common and specific unique (A, B and C) sRNAs and total (D, E and F) sRNAs between different libraries. (G) Known miRNAs among different libraries. [file 12870_2015_427_MOESM3_ESM.pdf]

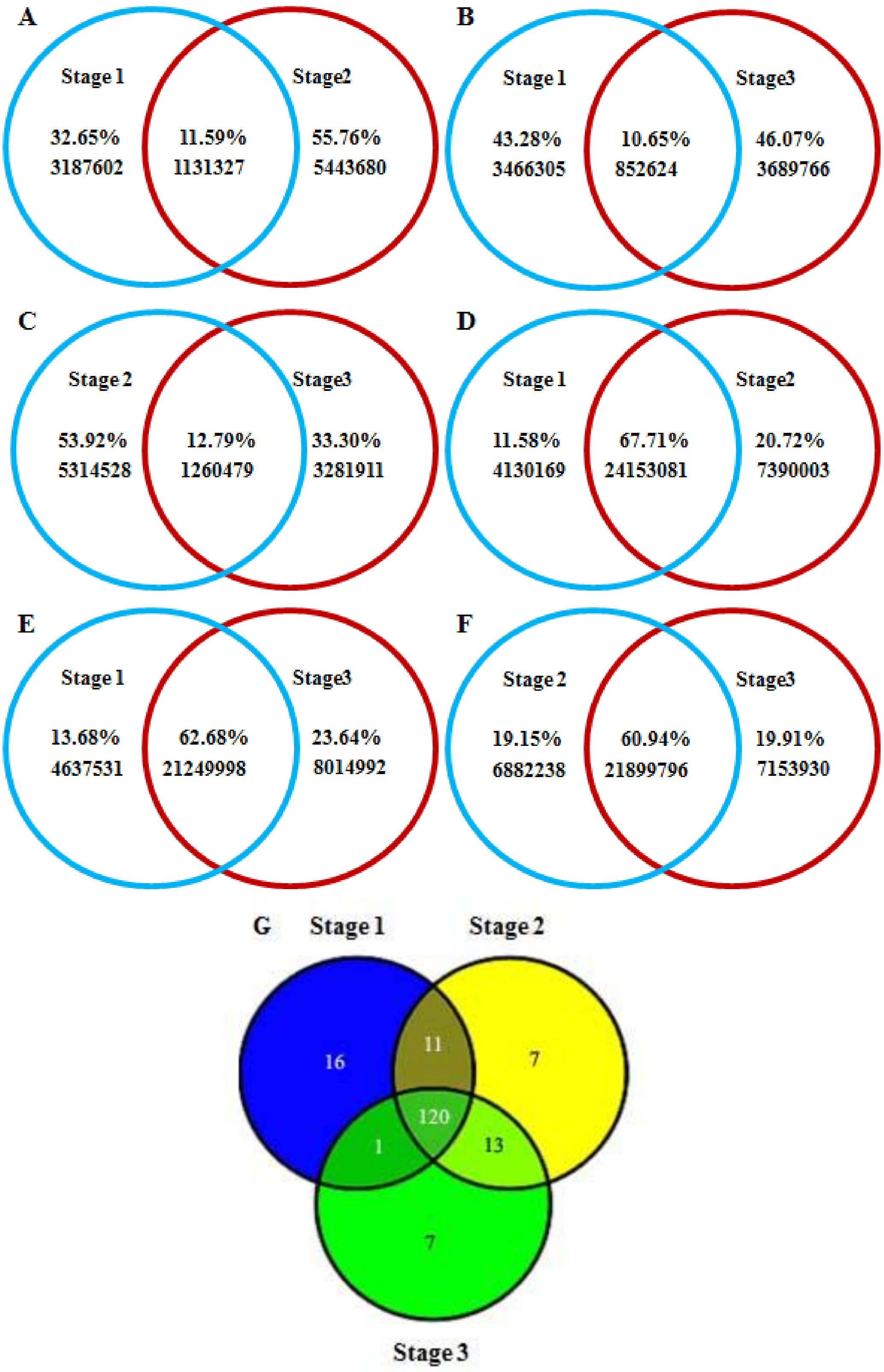

Additional file 3. Venn diagrams for analysis of Small RNAs. (A-F) Summary of common and specific unique (A, B and C) sRNAs and total (D, E and F) sRNAs between different libraries. (G) Known miRNAs among different libraries.
